# Uterine Hemorrhages

**Published:** 1894-01

**Authors:** Wm. Keiller

**Affiliations:** F. R. C. S., Ed.; Prof. of Anatomy, University of Texas; Galveston


### TEXAS MEDICAL JOURNAL.

ESTABLISHED JULY, 1885.

PUBLISHED MONTHLY.—SUBSCRIPTION $2.00 A YEAR.

VOL. IX. AUSTIN, JANUARY, 1894. NO. 7.

### Original Contributions

For Texas Medical Journal.

### UTERINE HEMORRHAGES.

BY WM. KEILLER, F. R. C. S., ED.
Prof. of Anatomy, University of Texas.

Read befre the Galveston County Medical Society, December 4, 1893.

GENTLEMEN:—Hemorrhage from the uterus (exclusive of those hemorrhages, more directly associated with obstetrics) is a symptom of so many conditions, and one of such great interest alike to the general practitioner and the specialist, that I do not think you will grudge me the time during which I shall direct your attention to the subject, even should I only succeed in making nothing more than a review, in marshaling before you systematically the whole army of diseased conditions which must be presented to the mind of the experienced physician by the symptom "uterine hemorrhage." It is perhaps a good business maxim to take every man for a knave till you find him out to be an honest man, and it is certainly a good rule in gynecology to consider every adult female as actually pregnant, or as having been so, till you have indubitable proof that it is otherwise. Bearing this rule in mind, and remembering also that uterine hemorrhage is always a symptom only, never a disease, we shall be in a proper frame of mind to approach the subject.

1. Profuse menstruation in the young unmarried woman, is not by any means an infrequent or unimportant condition.

No doubt careful examination in such cases would be instructive; but physical examination is not justified unless absolutely necessary, and most cases will yield to the systematic use of remedies directed towards improvement of the blood and relief of constipation. 5 grs. of dried sulphate of iron in capsule thrice daily after meals, and Hunjari Janos water every morning before breakfast, or for poorer patients sufficient sulphate of magnesia well diluted, in the morning, to move the bowels freely, will give relief, or if that fail ergot hamamelis, or hydrastis canadensis may be used to limit the discharge, while the former remedies are used between the periods.

### ABORTION.

Perhaps the most frequent class of cases that one meets with in practice is as follows:

Patient complains of being very frequently unwell and very copiously, though she used to be regular. Careful inquiry elicits that she was quite regular till some time previously, when her period was delayed for one, two, or more weeks, and then came rather profusely. She did not think herself pregnant, and does not think she had aborted. Since then menstruation has been frequent and profuse. Bimanual examination reveals some uterine enlargement, and often retroflexion, or version; nothing abnormal in the fornices vaginæ. Diagnosis, abortion followed by endometritis. Scrapings from such a uterus may consist of weak œdematous granulation tissue, or of excessively vascular mucous membrane with greatly complicated and distended uterine glands.

### TREATMENT.

Reduction of any retro-displacement that may exist, and the use of a suitable pessary with hot douches, laxatives, and 30 drops of fluid extract of ergot, ergotin 4 grs. in pill, or hamamelis, or hydrastis canadensis should be given a fair trial, as a certain number of the less severe cases will yield to such treatment; and we should not forget that while we may curette 99 times without trouble, in the 100th case we may have the most serious inflammatory complications. But if after fair trial these remedies fail, then there is nothing so safe or so sure as the curette. Like Volkman's spoon it is diagnostic as well as curative, as even the sharp curette in careful hands will not remove healthy mucous membrane. Dilate if necessary, curette, and after carefully removing all debris, swab out with liquified carbolic acid or iodised phenol. I do not think it matters whether an iodoform gauze drain is used or not—I prefer not, and think it unnecessary. In this connection I would like to emphasize the necessity of rigid antiseptics, and at least one week's rest in bed, with medical supervision in even the simplest cases, and I would prescribe ergot for a fortnight after operation.

But one is often called in for uterine hemorrhage due to incomplete abortion; and here it may be laid down as a rule that in a married woman previously regular, a sudden attack of profuse hemorrhage is in all probability due to an abortion, whether she thinks herself pregnant or not; for the most experienced woman may be thoroughly convinced that she is not pregnant, and yet you may find a two or three months' conception in process of expulsion. The diagnosis of such a case should offer little difficulty, and the indication is the speedy and thorough aseptic emptying of the uterus.

If the whole ovum, or the placenta, be in process of expulsion, sublimate douche, followed by a vaginal tampon of iodoform gauze, and ergot internally, will usually result in the complete emptying of the uterus in 24 hours, when the plug is to be removed; the ovum being found in the vagina. No case should be allowed to go on bleeding under expectant treatment. If plugging fails, the uterus should be thoroughly emptied by the finger or curette, under chloroform, washed out, and the patient kept in bed for a week.

### EXTRA-UTERINE PREGNANCY.

Now, I wish to put side by side with these cases of hemorrhage, due to abortion, a very different class of case, but one which may resemble it very closely. I mean ectopic pregnancy; and I consider this a matter of the utmost importance, as the last condition may be mistaken for abortion, and the mistake may lead to the gravest consequences. Think for a moment of the frequent course of an ectopic gestation.

Patient previously regular, misses a period. About the end of the first month she has a sudden severe pain in the ovarian region. This is followed by uterine 'hemorrhage more or less severe, and the *passage of decidua*, and while the decidua may go unnoticed altogether, it may be observed and mistaken for an abortion. The pain in the side was probably due to a sub-peritoneal rupture of the pregnant tube, but there may be no more trouble there, and the pain itself be entirely forgotten by the patient, and only to be elicited in her history by careful leading questions. Let the pain be forgotten, and your history is a complete one of an abortion, and the indication is the curette. One thing only remains to guide you past the quicksand, and that is the presence of a tumour on one side of the uterus. Miss that and you will make a fatal mistake, and may lose your patient. What, then, are the symptoms of ectopic pregnancy? First, the ordinary signs of pregnancy (all of which may be absent); second, the presence of a slowly-growing tumor, displacing the uterus to one side; third, the passage of decidua and irregular hemorrhages; fourth, paroxysmal pain on one side. The two essential factors in the diagnosis are the presence of the characteristic tumor and the passage of decidua. As regards treatment, there is now no difference of opinion as to its early removal by abdominal section. If it have ruptured it must be removed at once, if the patient be not too collapsed for operation; if diagnosed before rupture, it should be removed before rupture. The earlier it is undertaken the easier will be the operation.

Chronic cophoritis, and its common result, the small cystic ovary, dermoid tumors, and pyo-salpynx, may all have menorrhagia and irregular menstruation (rarely metrorrhagia) as a symptom. Of these a dermoid tumor, a pyo-salpynx, and a tubal gestation, may present difficulties in differentiation, but the treatment is the same for each condition, and it is notorious that when one opens the abdomen, one must be prepared for anything, often for what one least expects. It is to be remembered that any of these conditions may lead to persistent uterine hemorrhage, and that curetting the uterus for hemorrhage, due to a pyo-salpynx, has before now led to death by rupture of the tube; and the moral is this, "Never proceed to curette before you have carefully examined your patient bimanually after she is under chloroform, no matter how confident you are of your diagnosis." I remember two cases of my own which impressed me very strongly with the extent to which one may be misled by examination, and how the anæsthetic may clear up the diagnosis. The first was in a young woman who had never borne children. She was nervous, tender, and had well-braced abdominal muscles; and any one experienced in bimanual examination will appreciate how such a combination of conditions may make diagnosis impossible. But this case impressed me specially, because neither I myself, nor an experienced gynecological specialist, could detect a large uterine fibroid which, under chloroform, became so evident as to be child's play to the merest tyro. The second case was that of a multipara, with fatty abdominal walls. A miscarriage was followed by obscure pevlic symptoms, accompanied by high temperature. Careful examination, without anæsthesia, detected nothing in the pelvis. Several times I thought of giving her chloroform to examine again, but put it off. She ended by discharging an enormous quantity of pus at her umbilicus. This was treated by a drainage tube, and she made a good recovery. I have no doubt that examination made under an anæsthetic would have led to detection of fluctuation, and the opening of the abscess, a long time before it bursted.

It will be noted that I have been led by association of symptoms to follow abortion, as a cause, by an enumeration of causes external to the uterus itself. Let me now repeat that apart from heart, liver, and kidney affections, which, of course, must not be lost sight of, the conditions outside the uterus itself, which most frequently give rise to menorrhagia and metrorrhagia, are extra-uterine pregnancy, oophoritis, and small cystic ovaries, and pyo-salpynx. Returning to the uterus itself, metritis, subinvolution, endometritis, fungous endometritis, endocervicitis, all of which are conditions closely associated with each other, and all of which are usually, though not always, results of abortion, or parturition, are to be treated much as I have suggested under the heading of abortion; but the full treatment of these conditions is quite beyond the scope of my present paper.

### UTERINE OR CERVICAL POLYPUS.

A small uterine or cervical polypus is by no means an uncommon affection, and may give rise to troublesome hemorrhage. The currette may miss it entirely, and the diagnosis be impossible without cervical dilatation to the extent of admitting the finger. It is to be removed by polypus forceps.

### FIBRO-MYOMA.

Apart from the sequelæ of abortion or child-birth, there is no more common cause of menorrhagia and metrorrhagia than uterine fibroids, the interstitial and submucous varieties giving this form of trouble. The patient usually complains of pain, more or less constant, in the back, or ovarian regions, perhaps a sense of weight, and menorrhagia, or frequent menstruation, more seldom of metrorrhagia. The cavity of the uterus is enlarged, and possibly tortuous, causing a good deal o difficulty in passing a probe; bimanual examination reveals one or more rounded tumors continuous with the body of the uterus. With regard to treatment: Judging from the frequency with which one finds fibroids post mortem, the majority of cases give rise to no more trouble than some backache—are probably never brought before the physician. The amount of the hemorrhage does not depend on the size of the tumor, but on its relation to the uterine mucosa. Hemorrhage is usually the only symptom that requires treatment, for though they may give rise to hydronephrosis from pressure on the ureter, bladder irritation, or rectal symptoms, such cases are the exception, and not the rule; and most patients with fibroids will lead fairly comfortable lives, if you will keep their bowels regular. Of those cases complaining of hemorrhage, the majority will yield to ergot (or other uterine hemostatics—ergotine often acts better than the fluid extract) used persistently both at and before the period (30-drop doses of the fluid extract, or 4 grs. of the ergotine, thrice daily), combined with iron and laxatives. If this fail, oophorectomy has thoroughly stood the test of experience, but is not often possible in tumors larger than a three months pregnancy. Electricity has fallen very much into disfavor. No one now expects it to do more than relieve symptoms; never to dispel the tumor. But in interstitial fibroid, the galvanic current (positive or styptic pole used in the uterus) may, temporarily at least, stop the hemorrhage. Removal of the tumor (either by enucleation in suitable cases, or by excision of the uterus in whole or in part), is again regaining favor in many directions, but is only to be undertaken where hemorrhage or pressure threatens life.

Before leaving this subject, I would say two things: 1st, that as the majority of fibroids give rise to no trouble whatever, if the physician discover one by chance, he should not inform the patient of its presence, as it will only make her uneasy to no purpose, and, 2d, that any one with a fibroid may be at any time attacked by a severe hemorrhage, and that such a hemorrhage is to be treated by a uterine or vaginal tampon of iodoform gauze, followed by the curette when it is checked. This will temporarily stop it, and give time for other therapeutic measures.

INVERSION OF THE UTERUS.

The uterus may, usually following parturition, or as the result of an effort at expulsion of a peduncalated fibroid, be inverte or wholly or in part turned outside in. Again hemorrhage is a symptom, and vaginal examination reveals something very like a peduncalated fibroid in process of birth, but the hand on the abdomen will detect the absence of the inverted fundus. Such a condition, of course, must be treated appropriately. Recent cases are fairly easy to replace, older cases may present great difficulties.

SARCOMA AND CARCINOMA.

No disease comes on more insidiously, none will more readily escape detection, than cancer of the uterus, and in none is early diagnosis of more vital importance. It is here unnecessary to differentiate between sarcoma and carcinoma. Pain, a symptom often associated with cancer elsewhere, is here often absent till late, and a profuse watery discharge, accompanied by a little bleeding, is usually the first symptom noticed. Here the most important thing that I can say, is that no irregular hemorrhage should be attributed to the change of life, without the most careful examination. If a woman have ceased menstruating, and her periods return again, one should at once be suspicious of cancer, and if there be any doubt, scrapings from fundus or cervix should be examined microscopically, and the diagnosis thus made certain. Carcinoma, though most frequent in later life, is confined to no age—is found in extreme youth, and in the old. If in a supposed endometritis bleeding recur after repeated curettings, sarcoma of the body should at once be suspected, and the scrapings exanined,—indeed, it would be a good rule to examine microscopically the scrapings every time one curettes.

Mrs. K., aged 58; has had thirteen children—no miscarriages—last child eleven years ago. Ceased menstruating six years ago; had never seen anything till three months before consultation. One week before that she fell down stairs and a fortnight later noted a yellowish watery discharge, soon followed by blood. Since her fall she has had constant pain night and day in back and radiating round to the front of the abdomen on both sides. Pain of a griping, gnawing character, sometimes like labor pains, uterus very slightly enlarged, freely movable, os appeared only catarrhal. Scraping from cavity showed a very vascular rapidly growing columnar carcinoma. I removed the uterus per vaginam, clamping the broad ligament with pressure forceps, her narrow senile vagina making ligature impossible. Clamps removed in twenty-four hours. No hemorrhage. Patient made an excellent recovery and had good health and freedom from pain for six months, but then the disease returned in the vaginal roof and she died soon after.

Was the operation a failure? I feel sure it added six months of apparent health to her life span, and so far as we could judge there was a chance of a complete cure. I certainly consider the operation a justifiable one.

With reference to the treatment of cancers, in epithelioma of the cervix where the whole disease can be removed by supra-vaginal amputation of the cervix, that operation is to be preferred to hysterectomy, as the results are exceedingly good and the risks very small., Where the disease has gone farther than can be reached by this method, but has not spread to broad ligaments, vaginal walls or bladder, vaginal hysterectomy by ligature, or clamp, if the ligature be impossible, is the proper treatment. The results are as good as for cancer of the mamma, most cases having at least a period of comparative health.

Where the complete removal of the disease is impossible, hemorrhage and the fœtor of the discharge may be kept in check by the curette or thermocautery and iodoform tampons.

